# Decision-making of adjuvant therapy in pT1N1M0 gastric cancer: Should radiotherapy be added to chemotherapy? A propensity score-matched analysis

**DOI:** 10.7150/jca.52123

**Published:** 2021-01-01

**Authors:** Siwei Pan, Songcheng Yin, Zhi Zhu, Funan Liu, Huimian Xu

**Affiliations:** 1 Department of Surgical Oncology, the First Affiliated Hospital of China Medical University, Shenyang, China.; 2 Department of Surgical Oncology and General Surgery, Key Laboratory of Precision Diagnosis and Treatment of Gastrointestinal Tumors, Ministry of Education, The First Affiliated Hospital of China Medical University, Shenyang, China.; 3 Center for Digestive Disease, The Seventh Affiliated Hospital of Sun Yat-sen University, Shenzhen, China.

**Keywords:** early gastric cancer, lymph node metastasis, adjuvant therapy, radiotherapy, propensity score-matched analysis

## Abstract

**Background:** Early gastric cancer (EGC) with metastatic lymph nodes (mLNs) has a relatively higher recurrence rate and poorer prognosis than EGC without mLNs. However, the postoperative treatment directions of pT1N1M0 vary from different guidelines. This study attempted to confirm the value of postoperative treatments in pT1N1M0 GC patients.

**Methods:** Overall, 379 patients with pT1N1M0 GC following gastrectomy from 2000 to 2016 were selected from the Surveillance, Epidemiology, and End Results (SEER) database. Propensity score-matched (PSM) analysis was used to reduce bias. Overall survival was analyzed by Kaplan-Meier method and the log-rank test. Cox proportional hazards regression analyses were used to confirm the independent prognostic factors.

**Results:** Before matching, the results of survival analyses indicated that adjuvant chemotherapy (ACT) and chemoradiotherapy (ACRT) could significantly prolong the survival time of the cohort (*P <* 0.05). After PSM analysis, 136 patients remained and ACRT maintained significance in the survival analysis (*P =* 0.018). Furthermore, patients with well or moderately differentiated GC (HR = 0.226, *P =*0.018) or intestinal type GC (HR = 0.380, *P =* 0.040) achieved a significantly superior prognosis with ACRT, compared to patients receiving ACT.

**Conclusion:** The survival benefit of ACRT and ACT for pT1N1M0 GC patients following gastrectomy was confirmed in the SEER cohort. RT added to ACT might be recommended according to Lauren's classification and tumor grade in clinical decision making.

## Introduction

Early gastric cancer (EGC), in which tumor invasion is restricted to the mucosa or submucosa (T1), has a significantly more favorable prognosis than advanced gastric cancer (GC), with a 5-year survival rate of over 90% following radical resection 1, 2. Approximately 2.2%-7.0% of patients with EGC have been reported to experience recurrence after gastrectomy 2-5. However, patients with metastatic lymph nodes (mLNs) had a relatively higher recurrence rate and much poorer survival rate 1, 4, 6.

In the American Joint Committee on Cancer (AJCC) Cancer Staging Manual, and the third English edition of the Japanese Classification of Gastric Carcinoma (JCGC), pT1N1M0 was identified as stage IB with one or two mLNs 7, 8. It was demonstrated that pT1N1M0 GC only occupied 1.2%-13.1% of all patients, but 47%-73% of EGC with metastatic lymph nodes in previous studies 9-11. According to the National Comprehensive Cancer Network (NCCN) treatment guidelines, patients with mLNs should be treated with postoperative adjuvant chemotherapy (ACT) or chemoradiotherapy (ACRT) 12. In contrast, it is directed only observation for p-Stage I patients in Japanese gastric cancer treatment guideline, which including pT1N1M0 13. Furthermore, there was no direction showed in guidelines as to which patients should receive radiotherapy (RT) added to ACT.

Moreover, a previous study reported that there was no survival benefit from ACT or ACRT in pT1N1M0 patients 10. However, Priscilla *et al.* indicated, based on subgroup analyses, that patients with N1 showed a significant benefit from ACRT, compared to ACT 14, 15. The Adjuvant Chemoradiotherapy in Stomach Tumors (ARTIST) trial also indicated that ACRT prolonged survival in patients with positive lymph nodes, compared to ACT 16. Although the role of RT remains controversial, a series of studies have confirmed that postoperative adjuvant RT (ART), in addition to surgery, could improve overall survival (OS) in patients, or in those with positive surgical margins 17-20. Moreover, as a locoregional treatment, ART can control locoregional recurrence, which accounts for 17%-37% of all recurrences. The toxicity and side effects are also relatively mild compared to systemic CT 2, 3, 9.

Consequently, given the discrepancies among the guidelines and conclusions from previous studies, the current retrospective analysis was performed to define adjuvant therapy and value of RT in patients with pT1N1M0, and determine the factors guiding the selection of treatment options using the Surveillance, Epidemiology, and End Results (SEER) database.

## Materials and methods

### Patient Source

The SEER program collected and published incidence and survival data based on cancer registries, and covers approximately 26% of the US population 21, 22. The cohort for the current study was selected from the SEER database using SEER-stat software (SEER*Stat 8.3.6).

**Figure 1:**
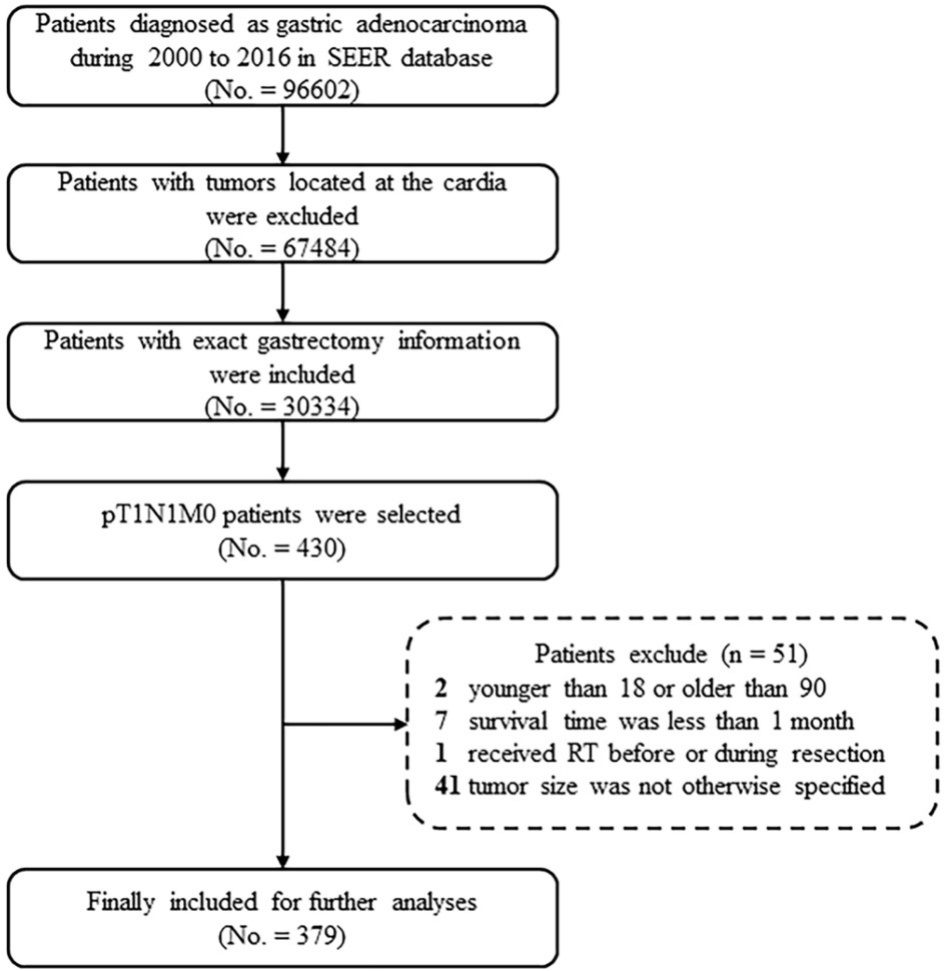
Case screening process for the current analyses from the SEER database. Abbreviations: SEER, Surveillance, Epidemiology, and End Results; RT, radiotherapy.

Patients who underwent gastrectomy and were subsequently diagnosed with gastric adenocarcinoma [*International Classification of Disease for Oncology, Third Edition* () code in the range of 8000-8152, 8154-8231, 8243-8245, 8250-8576, 8940-8950, and 8980-8990] from 2000 to 2016 were considered for this study. GC invading into the mucosa (T1a) or submucosa (T1b), and that were accompanied by one or two mLNs (N1), and no other metastases (M0), were selected. Moreover, patients with tumor locating at the cardia or esophagogastric junction were excluded. Patients were also excluded based on the following criteria: (1) younger than 18 or older than 90; (2) the clinical or follow-up information were not clear; (3) the survival time was less than 1 month; and (4) received RT before or during resection. After applying these criteria, 379 patients were included in the study (**Figure 1**).

The following demographic and pathological characteristics were extracted from the SEER database: race, sex, year of diagnosis, age, grade, the primary location and size of tumor, depth of invasion, number of examined lymph nodes (eLNs), positive LNs (pLNs), RT and CT situation, survival information at the last follow-up (Nov. 2018). Furthermore, Lauren's classification was also categorized by histological type, including diffuse type (ICD-O-3 codes: 8020-8022, 8142, 8145, and 8490) and intestinal type (ICD-O-3 codes: 8140, 8144, 8210-8211, 8260, and 8480-8481), which was also used in several previous studies 23-25.

### Statistical analysis

OS, defined as the survival time from radical resection to death. The Kaplan-Meier method was applied to calculated OS and verified the reliability by the log-rank test. The categorical variables were described as counts and proportions, and were compared using the Pearson's Chi-square test or the Fisher's exact test. Cox proportional hazards regression models were applied to identify the prognostic factors in univariate and multivariable analyses, with the following cofactors: sex, age, race, primary site and size of tumor, grade, Lauren's classification, T stage, number of eLNs and pLNs, and adjuvant therapy types.

Because the data from the SEER program were not assigned randomly, and due to the imbalanced covariates between the RT and non-RT groups, a propensity score-matched (PSM) analysis was applied to reduce the effect of possible confounding and selection bias in the two subgroups 26, 27. A 1:1 matching without replacement was completed using the nearest-neighbor match on the logit of the propensity score within a caliper of 0.1, which was derived based on sex, age, race, primary site, size, Lauren's classification, grade, number of eLNs and pLNs, and the patients' situations of receiving ACT.

Statistical analyses were carried out using R software (version 3.5.3; R Foundation for Statistical Computing, Vienna, Austria) and SPSS (version 23.0; SPSS Inc, Chicago, IL). A two-tailed *P*-value < 0.05 was considered statistically significant in all analyses.

## Results

### Clinicopathologic Characteristics of the Overall Cohort

The demographic and pathological characteristics of 379 patients are illustrated in **Table 1**. 141 (37.2%) patients underwent pRT, while 238 (62.8%) did not. The median follow-up time was 43 months. The median age was 71-years-old (IQR, 62-78 years) and most patients (290, 76.5%) were over 60-years-old. Based on Lauren's classification, ~20% of patients (84, 22.2%) were identified as having diffuse type GC. Additionally, the median tumor size overall was 2.6 cm. Sixty-eight (17.9%) patients were at T1a stage, while 311 (82.1%) were T1b. For adjuvant therapy types, ~50% of patients (185, 48.8%) receiving only observation, 15 (4.0%) receiving only ART, 53 (14.0%) receiving only ACT and 126 (33.2%) receiving ACRT. In the patients receiving RT and not RT groups, they were not assigned randomly according to the following characteristics: age, pLNs and the situations of receiving ACT (*P <* 0.05).

### Overall Survival and Cox Proportional Hazards Regression Analysis

**Figure 2:**
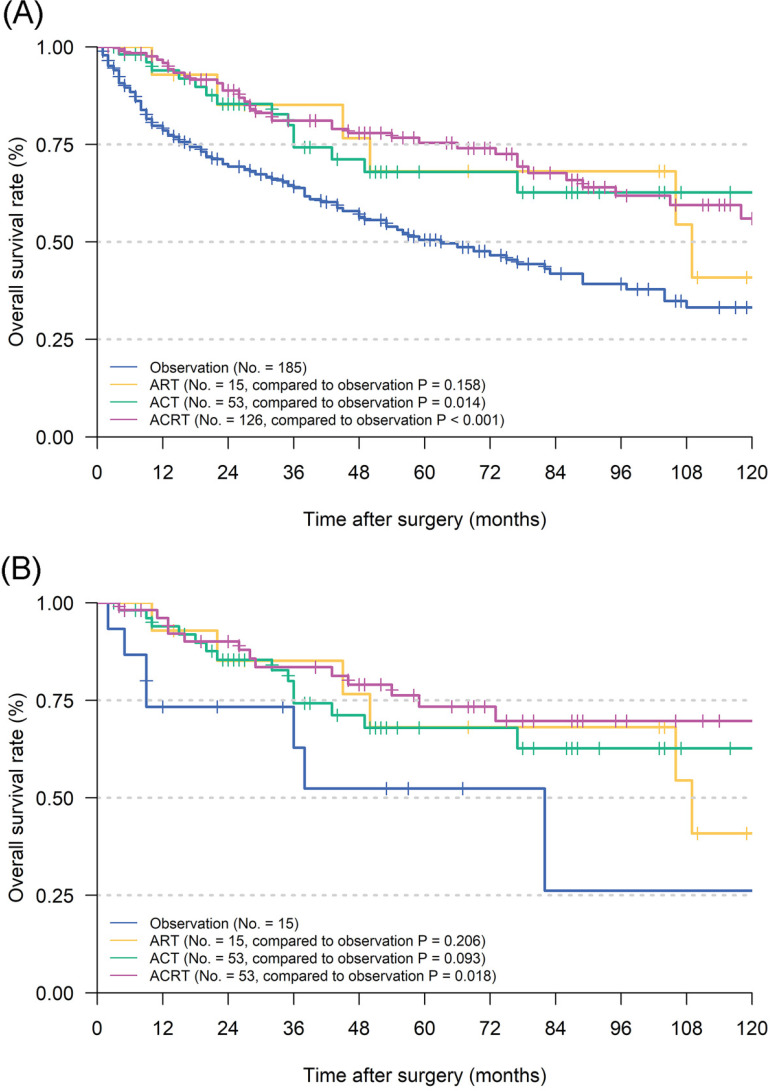
Kaplan Meier overall survival curves of pT1N1M0 gastric cancer patients according to different adjuvant therapy types. **A.** The whole cohort; **B.** the cohort after PSM analysis. Abbreviations: ART, adjuvant radiotherapy; ACT, adjuvant chemotherapy; ACRT, adjuvant chemoradiotherapy.

Before matching, univariate analyses illustrated that age, Lauren's classification, grade, eLNs and adjuvant therapy could significantly affect the OS of patients (**Table 2**). Cox proportional hazards regression analyses confirmed that eLNs (*P =* 0.007) and adjuvant therapy (*P =* 0.002) could still improve OS in the multivariate analyses and played a role as independent prognostic factor. Furthermore, survival analyses indicated that postoperative ACT (*P =* 0.014) and ACRT (*P <* 0.001) could significantly prolong the survival of patients when compared to observation (5-year OS: 50.5%), while ART not (*P =* 0.158) (**Figure 2A**). To confirm a balanced baseline of patients' characteristics, PSM analysis was performed for patients who received RT versus those that did not (**Table 3**). It was determined that 68 pairs of matched patients lacked a significant difference in the characteristics between the two groups (overall *P* > 0.05 based on Person Chi-square or Fisher exact tests). Through survival analyses, ACRT (*P =* 0.018) still kept significance in the matched cohort when compared to observation (5-year OS: 52.4%), while ACT (*P =* 0.093) and ART not (*P =* 0.206) (**Figure 2B**).

### Survival and subgroup analysis between adjuvant chemotherapy and chemoradiotherapy

**Figure 3:**
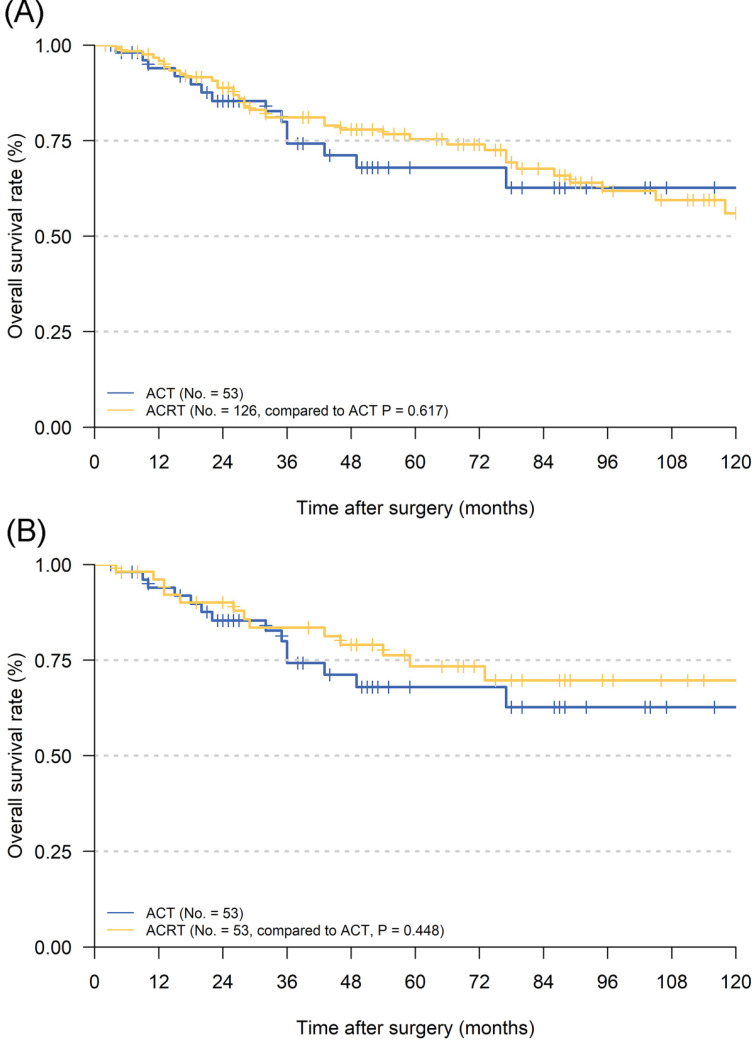
Kaplan Meier overall survival curves of pT1N1M0 gastric cancer patients whether to add radiotherapy to adjuvant chemotherapy. **A.** The whole cohort; **B.** the cohort after PSM analysis. Abbreviations: ACT, adjuvant chemotherapy; ACRT, adjuvant chemoradiotherapy.

**Figure 4:**
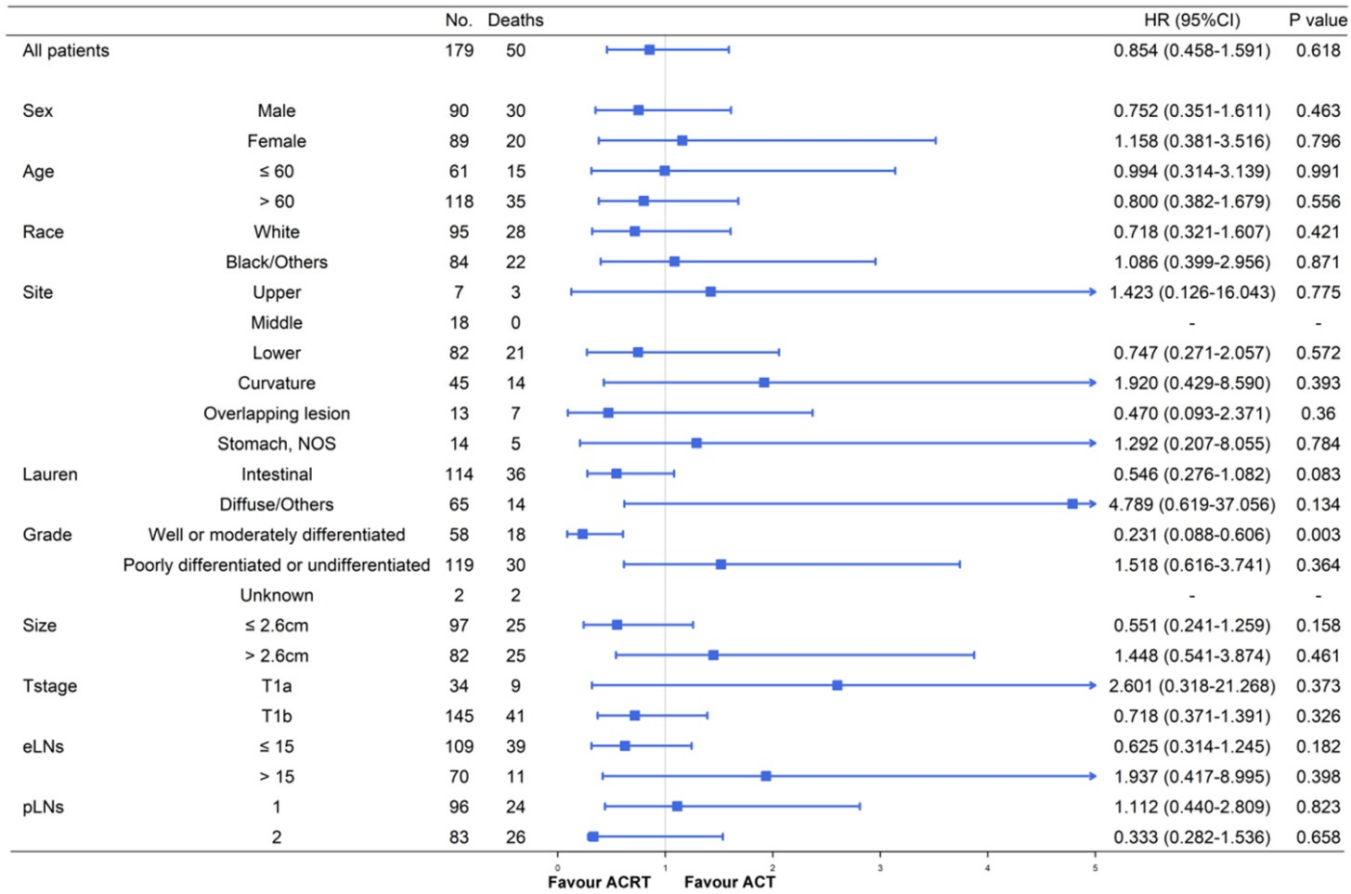
Subgroup analyses and forest plot of HRs and 95% CIs for overall survival of the whole cohort receiving ACT and ACRT. Abbreviations: No., number of patients; HR, hazard ratio; CI, confidence interval; NOS, not otherwise specified; eLNs, examined lymph nodes; pLNs, positive lymph nodes.

**Figure 5:**
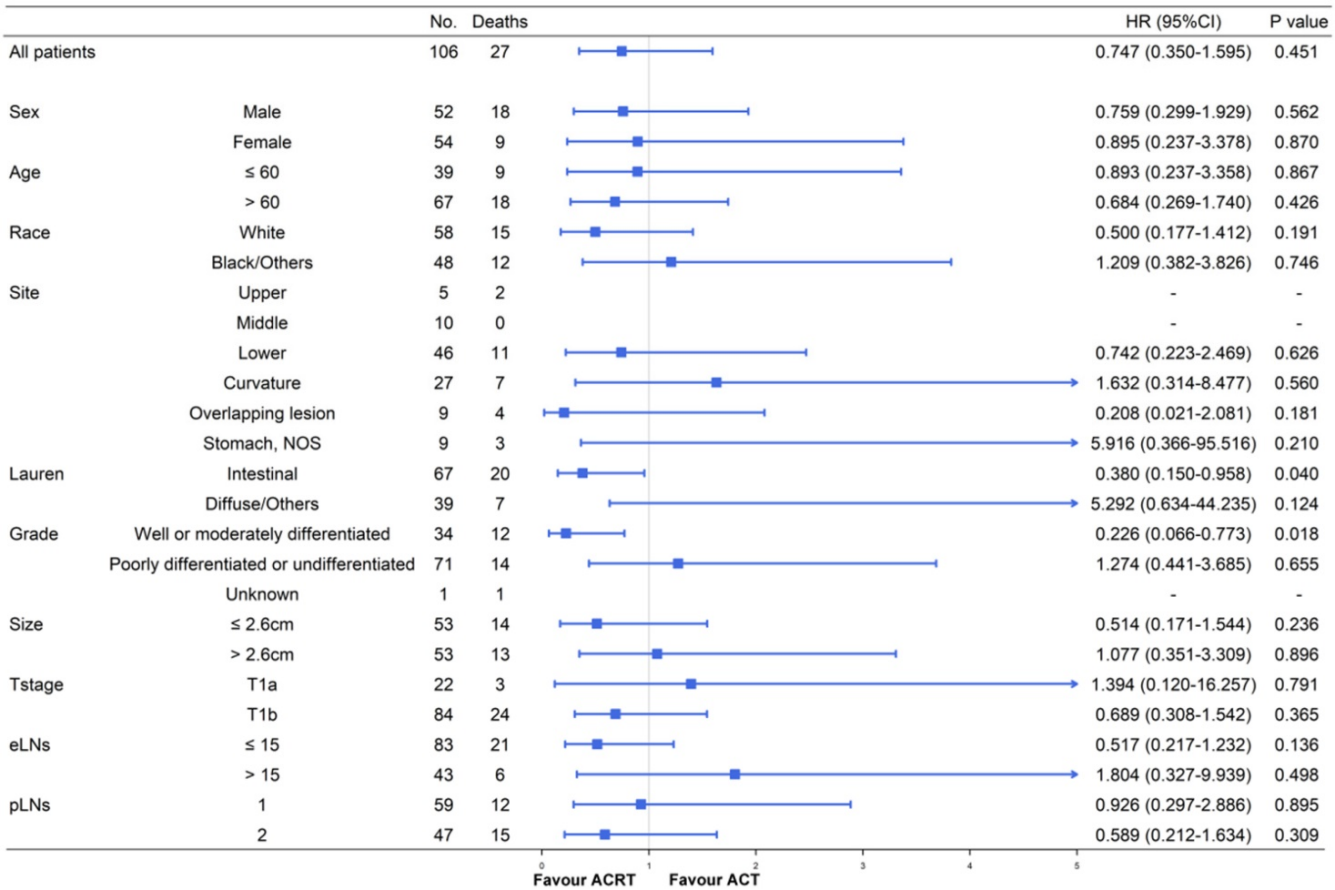
Subgroup analyses and forest plot of HRs and 95% CIs for overall survival of the cohort receiving ACT and ACRT after PSM analysis. Abbreviations: No., number of patients; HR, hazard ratio; CI, confidence interval; NOS, not otherwise specified; eLNs, examined lymph nodes; pLNs, positive lymph nodes.

Although ACT did not show significant survival improving in the matched cohort when compared to observation, there were also no significant difference in survival impact when compared ACT and ACRT in the matched (5-year OS: 67.9% vs. 73.3%, *P =* 617) or non-matched (5-year OS: 67.9% vs. 75.4%, *P =* 0.448) cohort (**Figure 3**). Herein, due to the survival impact of ACT and ACRT in patients with pT1N1M0 GC, we sought to determine the factor on whether it was necessary to administer RT. Exploratory subgroup analyses between ACT and ACRT groups before matched were performed for the following factors: sex, age, race, primary site, Lauren's classification, grade, size, T stage, eLNs and pLNs (**Figure 4**). We concluded that patients with well or moderately differentiated GC achieved significantly superior HR with ACRT than those that did not receive RT [HR = 0.231, 95% confidence interval (CI): 0.088-0.606, *P =* 0.003]. Otherwise, there was potential survival advantage for patients with intestinal type when received ACRT (HR = 0.546, 95% CI: 0.278-1.082, *P =* 0.083). After performing PSM analysis, the survival advantage of ACRT for patients with well or moderately differentiated GC was still significant when compared to those receiving ACT (HR = 0.226, 95% CI: 0.066-0.773, *P =* 0.018) (**Figure 5**). In addition, ACRT did prolong the survival of patients with intestinal type in the matched cohort (HR = 0.380, 95% CI: 0.150-0.958, *P =* 0.040). In contrast, the similar difference in OS was not illustrated in patients with diffuse or other type GC (HR = 5.292, 95% CI: 0.634-44.235; *P =* 0.124). Furthermore, there was a 26% increase in 5-year OS in patients with intestinal type GC after receiving ACRT than ACT (5-year OS: 75.1% vs. 59.1%, respectively). A more significant advantage could be observed in patients with well or moderately differentiated GC between ACRT and ACT (5-year OS: 71.8% vs. 31.1%, respectively). There was no significant survival difference in sex, age, race, site, size, T stage, eLNs and pLNs between ACRT and ACT, which indicated that former factors did not determine the application of RT.

**Figure 6:**
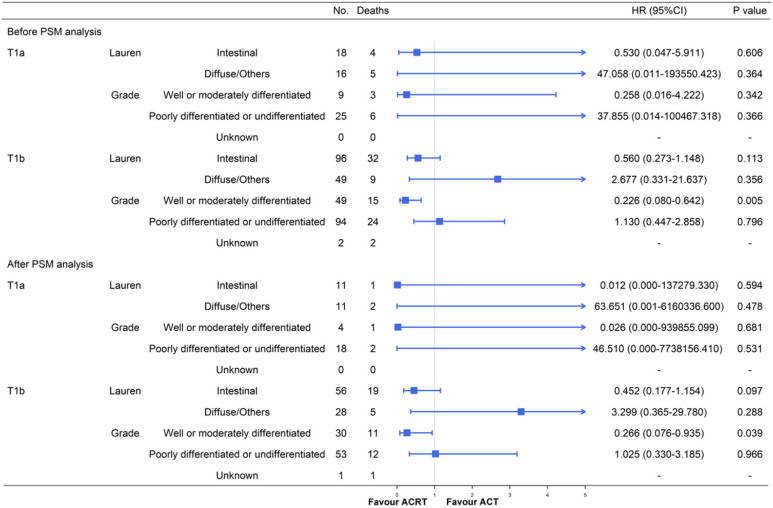
Subgroup analyses and forest plot of HRs and 95% CIs for overall survival of the T1a and T1b GC cohort receiving ACT and ACRT. Abbreviations: No., number of patients; HR, hazard ratio; CI, confidence interval.

Furthermore, due to the large population deviation between T1a and T1b stage, we also performed a subgroup analysis after separating them (**Figure 6**). The results of patients with T1b GC were mostly similar with the former conclusions either before or after the PSM. A significant advantage could be observed in T1b patients with well or moderately differentiated GC after receiving ACRT (before PSM: HR = 0.226, 95% CI: 0.080-0.642, *P =* 0.005; after PSM: HR = 0.266, 95% CI: 0.076-0.935, *P =* 0.039). The difference was that T1b patients with intestinal type GC did not gain a significant survival advantage from ACRT after PSM analysis (HR = 0.452, 95% CI: 0.177-1.154, *P =* 0.097) when the T1a and T1b combined cohort did (*P =* 0.040). We thought that the deviation of the results was caused by the small sample size of T1a patients and we decided to perform further prospective studies to expand the sample size and verify the conclusions.

## Discussion

In the present study, 379 post-gastrectomy GC patients were selected from the SEER database who were diagnosed with pT1N1M0 gastric cancer and evaluated the effect of adjuvant therapy on survival. In survival analyses, we confirmed that ACT and ACRT could significantly improve patients' prognosis. Moreover, we found that pT1N1M0 patients with well or moderately differentiated GC or intestinal type GC could benefit from ACRT while the others not.

GC patients with pT1 stage was confirmed as EGC who have been confirmed to have prominent 5-year OS and a low rate of recurrence 1-5. However, those with metastatic lymph nodes had a relatively higher recurrence rate, of which, pT1N1M0 patients accounted for 47%-73% 9-11. Considering postoperative treatment strategies, NCCN guidelines recommend ACT or ACRT for GC with any metastatic lymph nodes 12. In contrast, the Japanese gastric cancer treatment guideline only recommends observation for p-Stage I patients, including pT1N1M0 patients 13. Moreover, patients with a higher stage than T1N0 are recommended to undergo ACT or ACRT by the European Society for Medical Oncology (ESMO) guidelines 28. There were two confusing aspects among guidelines: 1) the different directions and 2) there was no exact information on which patients should receive ACT and which patients should receive ACRT.

A series of previous studies have demonstrated the beneficial effect of ACT or ACRT on survival for stage II or higher GC patients 29-32. Moreover, a SWOG-directed INT-0116 trial indicated that ACRT could achieve superior OS and relapse-free survival than surgery alone for stage II/III GC 32. MAGIC 31, ACTS-GC 30 and CLASSIC 29 trials also established an improvement in survival due to perioperative or postoperative ACT in cohorts with mostly stage II (or higher) patients. However, a Korean study failed to indicate a benefit from ACT or ACRT on survival and tumor recurrence in pT1N1M0 patients; thus, supporting the Japanese gastric cancer treatment guideline 10.

In the ARTIST trial, although no significant difference in survival was observed between ACT and ACRT in the overall cohort, superior survival benefits were observed from ACRT vs. ACT in lymph node-positive patients (HR = 0.700, 95% CI: 0.493-0.994) 15. A study by Priscilla *et al.* also indicated that N1 patients achieved a significant benefit from ACRT, compared to ACT in subgroup analyses 14.

So far, based on the conclusions above, the postoperative treatment for pT1N1M0 gastric cancer remains disputed. RT showed a significant benefit in OS and a decrease in locoregional relapse rate among patients undergoing resection 17-20. In an analysis of the National Cancer Database of America, a 5% significant advantage was observed in the 5-year OS, which was due to the administration of RT added to ACT (46% vs. 41%, respectively) 14. That study also revealed a significant survival improvement from preoperative, postoperative, and intraoperative RT in addition to resection in GC patients 19. Furthermore, locoregional recurrence, which accounts for 17%-37% of all recurrences in pT1N1M0 patients, might benefit from the locoregional control of RT. Thus, in addition to define appropriate adjuvant therapy type for different conditions, we sought to evaluate the effect of RT in pT1N1M0 patients.

In 379 pT1N1M0 GC patients who underwent surgery in the SEER database, no survival advantage was found in patients receiving ART alone, compared to observation (**Figure 2**). To the contrary, ACT and ACRT were both confirmed having superior ability to prolong OS in patients with pT1N1M0 GC than observation. Therefore, our conclusions supported that pT1N1M0 GC patients should receive at least ACT after gastrectomy. It also indicated that even in the case of EGC, systematic adjuvant therapy after operation can effectively relieve potential micro-metastases and uncleared lesions in the presence of mLNs.

To our best knowledge, although many studies had focused on the effectiveness of postoperative ACRT, most of them were compared with surgery alone 32-34. In pT1N1M0 GC patients, as an EGC, it is important to reduce unnecessary treatment as much as possible in order to avoid excessive medical treatment, reduce expenses and improve the quality of life. In the current study, we found that there was no significant increase in the 5-year OS between patients who received ACT and ACRT (**Figure 3**). However, there was still a ~5% increase in the 5-year OS in patients with ACRT than ACT. Therefore, we were determined to carry out subgroup analyses in order to find out which patients were suitable for RT in addition to ACT.

In further subgroup analyses (**Figure 4** and **Figure 5**), significant increases in survival were observed in the intestinal type GC following ACRT in the matched cohort. However, there was no significant survival benefit in diffuse or other type GC patients from ACRT, compared to patients not receiving RT. Furthermore, in both matched and non-matched cohorts, RT in addition to ACT had been confirmed to prolong survival in patients with well or moderately differentiated with lower HRs. Thus, the results of the subgroup analyses directed that we could focus on these two types of patients and applied RT on the basis of ACT to achieve a significantly better prognosis, while the other patients just receiving ACT in clinical decision-making.

Highly differentiated and intestinal types are good behaviors directing betted prognosis in most GC patients, which might indicate that the intensity of treatment could be reduced 35-37. However, the results of the current study were just the opposite in pT1N1M0 GC patients. We supposed that the contradiction might be due to some good prognostic behaviors in EGC patients with mLNs might indicate potential undiscovered malignant factors like micro-metastasis, isolated tumor cells, gene mutation, or etc. Therefore, this suggested that we should pay more attention to EGC patients with mLNs.

Compared to the NCCN and ESMO guidelines directing ACT or ACRT for pT1N1M0 GC patients, or the Japanese gastric cancer treatment guideline that suggest only observation in such patients, we found that it was necessary to perform adjuvant therapy in order to prolong the survival time of pT1N1M0 GC patients. When screening patients for performing ACRT or ACT, Lauren's classification and tumor grade were two confirmative factors that could direct whether to administer RT added to ACT. Furthermore, as a locoregional treatment, RT might have some toxic side effects. Especially among elderly patients with pT1N1M0 EGC, we prefer to clarify the specific pathological data of the patient and then make a decision on the treatment plan, based on their physical conditions.

The current study had several limitations. First, our analyses were based on retrospective data, and the selection principle was based on diagnosis, demographic and pathological characteristics, and other information existing in the SEER database. Thus, this approach might result in deviations due to various diagnoses or treatment principles among the multiple medical centers contributing to the database. Second, the sample size of the study was only 379 patients because of the specific disease stage, with 136 after PSM analysis, which was too small to be relied upon. In addition, the overall cohort was from the SEER database and the conclusions were mainly applicable to American patients. Thus, further analyses are required for Chinese or Asian patients.

## Conclusions

In summary, although a clear treatment plan for pT1N1M0 GC remains controversial, a survival benefit was confirmed for ACT and ACRT in pT1N1M0 GC patients following gastrectomy. Moreover, RT added to ACT could be recommended in clinical decision making according to the Lauren's classification and grade of pT1N1M0 GC. Only pT1N1M0 patients with well or moderately differentiated or intestinal type GC benefited from ACRT with 40.7% and 26.0%, respectively, increases in 5-year OS than ACT, while the others did not. In order to obtain solid evidence of the conclusions described in this study, a prospective randomized controlled study is required, after which, further analyses will settle the optimal indications of ACRT and ACT.

## Figures and Tables

**Table 1:** Demographic and pathological characteristics of the 379 patients in this study

Characteristic	All Patients (No. 379)		Radiotherapy (No. 141)		No Radiotherapy (No. 238)		*P* value^#^
	No.	%	No.	%	No.	%	
**Age of years**							**0.003**
≤ 60	89	23.5	45	31.9	44	18.5	
> 60	290	76.5	96	68.1	194	81.5	
**Race**							0.190
White	202	53.3	69	48.9	133	55.9	
Black/Others*	177	46.7	72	51.1	105	44.1	
**Sex**							0.803
Male	194	51.2	71	50.4	123	51.7	
Female	185	48.8	70	49.6	115	48.3	
**Primary Site**							0.245
Upper	13	3.4	4	2.8	9	3.8	
Middle	49	12.9	15	10.6	34	14.3	
Lower	179	47.2	69	48.9	110	46.2	
Curvature	81	21.4	37	26.2	44	18.5	
Overlapping lesion	26	6.9	9	6.4	17	7.1	
Stomach, NOS	31	8.2	7	5.1	24	10.1	
**Lauren**							0.242
Intestinal	261	68.9	92	65.2	169	71.0	
Diffuse/Others	118	31.1	49	34.8	69	29.0	
**Grade**							0.233
Well or moderately differentiated	150	39.6	48	34.0	102	42.9	
Poorly differentiated or undifferentiated	221	58.3	90	63.8	131	55.0	
Unknown	8	2.1	3	2.2	5	2.1	
**Size**							0.552
≤ 2.6 cm	191	50.4	77	54.6	114	47.9	
> 2.6	188	49.6	64	45.4	124	52.1	
**T stage**							0.454
T1a	68	17.9	28	19.9	40	16.8	
T1b	311	82.1	113	80.1	198	83.2	
**eLNs**							0.342
≤ 15	222	58.6	87	61.7	135	56.7	
> 15	157	41.4	54	38.3	103	43.3	
**pLNs**							**0.009**
1	239	63.1	77	54.6	162	68.1	
2	140	36.9	64	45.4	76	31.9	
**Adjuvant chemotherapy**							**<0.001**
Yes	179	47.2	126	89.4	53	22.3	
No/Unknown	200	52.8	15	10.6	185	77.7	
**Adjuvant therapy**							
Observation	185	48.8	-	-	-	-	
ART	15	4.0	-	-	-	-	
ACT	53	14.0	-	-	-	-	
ACRT	126	33.2	-	-	-	-	

No.: number of patients; NOS: not otherwise specified; eLNs: examined lymph nodes; pLNs: positive lymph nodes; ART: adjuvant radiotherapy; ACT: adjuvant chemotherapy; ACRT: adjuvant chemoradiotherapy. *Referring to American Indian/AK Native, Asian/Pacific Islander; #Categorical variable, Chi-square or Fisher test.

**Table 2:** Univariate and multivariate analysis of risk factors of pT1N1M0 gastric cancer patients before PSM analysis

Characteristics	Univariate analysis	Multivariate analysis		
	*P* value	HR	95% CI	*P* value
**Sex**	0.797			
Male				
Female				
**Age**	**0.002**			0.057
≤ 60		Ref		
> 60		1.591	0.987-2.565	0.057
**Race**	0.904			
White				
Black/Others*				
**Site**	0.834			
Upper				
Middle				
Lower				
Curvature				
Overlapping lesion				
Stomach, NOS				
**Lauren**	**0.023**			0.557
Intestinal		Ref		
Diffuse/Others		0.880	0.573-1.350	0.557
**Grade**	**0.006**			0.072
Well or moderately differentiated		Ref		
Poorly differentiated or undifferentiated		0.845	0.582-1.227	0.377
Unknown		2.216	0.918-5.350	0.077
**Size**	0.074			
≤ 2.6 cm				
> 2.6 cm				
**T stage**	0.779			
T1a				
T1b				
**eLNs**	**0.010**			**0.007**
≤ 15		Ref		
> 15		0.609	0.424-0.873	0.007
**pLNs**	0.133			
1				
2				
**Adjuvant therapy**	**<0.001**			**0.002**
Observation		Ref		
ART		0.577	0.249-1.336	0.199
ACT		0.614	0.345-1.091	0.096
ACRT		0.480	0.323-0.714	<0.001

HR: hazard ratio; NOS: not otherwise specified; eLNs: examined lymph nodes; pLNs: positive lymph nodes; ART: adjuvant radiotherapy; ACT: adjuvant chemotherapy; ACRT: adjuvant chemoradiotherapy. *Referring to American Indian/AK Native, Asian/Pacific Islander.

**Table 3:** Demographic and pathological characteristics of the 136 patients after propensity score-matched analysis

Characteristic	Radiotherapy (No. 68)		No Radiotherapy (No. 68)		*P* value^#^
	No.	%	No.	%	
**Age of years**					0.714
≤ 60	23	33.8	21	30.9	
> 60	45	66.2	47	69.1	
**Race**					0.607
White	32	47.1	35	51.5	
Black/Others*	36	52.9	33	48.5	
**Sex**					0.732
Male	33	48.5	35	51.5	
Female	35	51.5	33	48.5	
**Primary Site**					0.396
Upper	2	2.9	4	5.9	
Middle	7	10.3	7	10.3	
Lower	33	48.5	30	44.1	
Curvature	19	27.9	14	20.6	
Overlapping lesion	5	7.4	5	7.4	
Stomach, NOS	2	3.0	8	11.7	
**Lauren**					0.714
Intestinal	45	66.2	47	69.1	
Diffuse/Others	23	33.8	21	30.9	
**Grade**					0.842
Well or moderately differentiated	24	35.3	24	35.3	
Poorly differentiated or undifferentiated	42	61.8	43	63.2	
Unknown	2	2.9	1	1.5	
**Size**					0.493
≤ 3 cm	33	48.5	37	54.4	
> 3 cm	35	51.5	31	45.6	
**T stage**					0.396
T1a	16	23.5	12	17.6	
T1b	52	76.5	56	82.4	
**eLNs**					0.727
≤ 15	41	60.3	39	57.4	
> 15	27	39.7	29	42.6	
**pLNs**					1.000
1	40	58.8	40	58.8	
2	28	41.2	28	41.2	
**Adjuvant chemotherapy**					1.000
Yes	53	77.9	53	77.9	
No/Unknown	15	22.1	15	22.1	

No.: number of patients; NOS: not otherwise specified; eLNs: examined lymph nodes; pLNs: positive lymph nodes; ART: adjuvant radiotherapy; ACT: adjuvant chemotherapy; ACRT: adjuvant chemoradiotherapy. *Referring to American Indian/AK Native, Asian/Pacific Islander #Categorical variable, Chi-square or Fisher test.

## Abbreviations

EGC: early gastric cancer
GC: gastric cancer
mLNs: metastatic lymph nodes
AJCC: American Joint Committee on Cancer
JCGC: Japanese Classification of Gastric Carcinoma
NCCN: National Comprehensive Cancer Network
ACT: adjuvant chemotherapy
ACRT: adjuvant chemoradiotherapy
ART: adjuvant radiotherapy
SEER: Surveillance, Epidemiology, and End Results
eLNs: examined lymph nodes
pLNs: positive lymph nodes
PSM: propensity score-match
HR: hazard ratio
ESMO: European Society for Medical Oncology
